# Shortcomings in snake bite management in rural Cameroon: a case report

**DOI:** 10.1186/s13104-017-2518-8

**Published:** 2017-06-08

**Authors:** Frank-Leonel Tianyi, Christian Akem Dimala, Vitalis Fambombi Feteh

**Affiliations:** 1 Mayo Darle sub-Divisional Hospital, Mayo Banyo, Adamaoua Region Cameroon; 2 Orthopaedics Department, Southend University Hospital, Essex, UK; 3 Health and Human Development (2HD) Research Network, Douala, Cameroon; 4 Njikwa District Hospital, Momo, North West Region Cameroon; 5 Mayo Darle sub-Divivsional Hospital, Mayo Banyo, Adamawa region Cameroon

**Keywords:** Snake bite, Face, Severe, Anti-venom, Sub-optimal, Children, Cameroon

## Abstract

**Background:**

Snake bites are an important public health problem in developing countries with most bites occurring in rural areas. Severe envenomation often occurs in children and following bites to the face. Prompt administration of potent anti-venom remains the mainstay of management. However in Cameroon, the use of anti-venoms is limited by non-availability, high cost (where available) and poor mastery of treatment guidelines.

**Case presentation:**

We present a 10-year-old muslim Cameroonian child from an enclaved rural area, brought to the hospital 12 h after a snake bite to the face, with signs of severe envenomation. Despite the suboptimal anti-venom dose administered to this patient due to a stock out of this medication, supportive therapy was beneficial in ensuring a positive outcome and satisfactory recovery.

**Conclusion:**

This highlights snake bites as a public health problem due to the lack of snake anti-venoms in peripheral health facilities, rendering them unable to appropriately manage these cases. National health policies should encourage constant peripheral availability of anti-venoms and the institution of an intervention package for snake bite management, comprising: treatment protocol, staff training, monitoring of compliance and community education to help reduce the mortality and morbidity from snake bites.

## Background

Snake bite is an important but often neglected public health problem, common in tropical developing countries [1, 2]. Children are more frequently bitten compared to adults and are at risk of severe adverse effects due to their small sizes [3, 4]. Most bites often occur in rural settings where inhabitants usually cannot afford standard treatment which involves the use of appropriate anti-venoms [2, 5]. Coupled with the fact that most bites often occur at locations far-off from the hospital, making timely arrival unlikely. Moreover, local health facilities usually lack qualified personnel and equipment to implement life-saving interventions such as invasive ventilation, hemodialysis and inotropic support [6–8].

The lower and upper limbs are the most common sites for bites [9], with bites on the face being extremely rare [10]. Bites involving children and/or the face are considered as cases of severe envenomation [10] and usually require timely administration of an appropriate dose of an effective anti-venom [1], and optimal care in a hospital equipped to implement life-saving interventions to improve chances of survival [6]. However, in many developing countries, the use of anti-venoms is limited by non-availability, high cost [9] and poor mastery or absence of standardized guidelines on the utilization of anti-venoms where available [1]. In Cameroon, the epidemiological picture of this public health problem is worrisome, since a report in 2015 suggested no central store of wholesale medication suppliers possessed stocks of anti-venoms [11]. This survey of peripheral health facilities found that most of these health facilities were deficient in anti-venoms in their emergency kits [11]. Also, prognosis of some snake bite victims is further compounded by hindrances to early and safe referral [8].

We present the case of a 10 year old Cameroonian girl with late presentation to a local health center following snake bite to the face.

## Case presentation

A 10-year-old muslim Cameroonian girl with no significant past history, from an enclaved rural area of the Adamawa region of Cameroon, presented to our hospital with marked facial swelling and gross hematuria for about 8 h following a suspected snake bite while playing in the farm. She reported a sharp pain in the right temple followed by bleeding and worsening of the pain at the bite site. The snake was not seen but she was treated with topical and oral application of herbal concoctions. She passed out bloody urines roughly 4 h after the incident. Persistence of hematuria and progressive facial swelling prompted consultation in our hospital, located over 80 km away through deplorable roads. She arrived at our hospital roughly 12 h after the incident. On admission, there was no fever, no bleeding from the mouth or nostrils, no seizures, no loss of consciousness, no loss of vision or neurological manifestations.

**Fig. 1:**
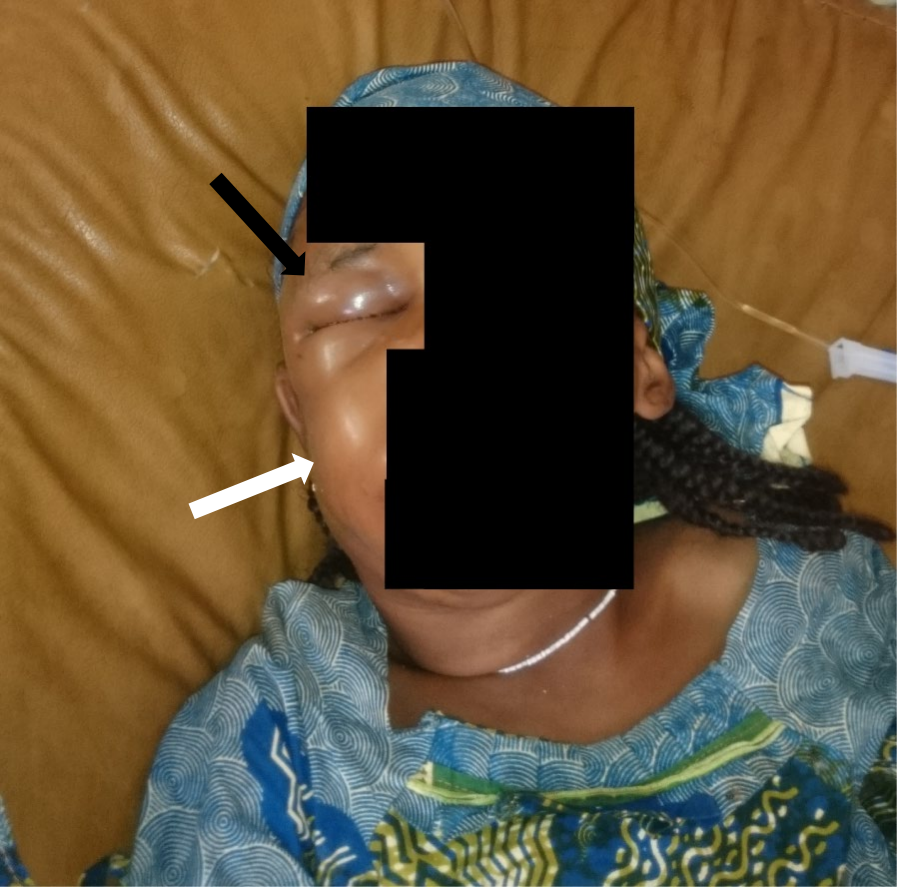
Facial swelling (*white arrow*) and marked right periorbital edema (*black arrow*) on admission

On examination, she was conscious and calm, with signs of mild respiratory distress. She had a temperature of 37.4 °C, pulse rate of 132 beats/min, regular with a full volume, respiratory rate of 31 breaths/min and she weighed 18 kg. There was a marked facial swelling with right peri-orbital edema (Fig. 1) and a bleeding single fang mark 2 cm anterior to the tip of the right ear. She had mildly pale conjunctivae, no jaundice, pupillary and ptosis assessment was not possible due to the severe facial swelling. She had mild nasal flaring and intercostal recession, vesicular breath sounds in both lung fields with no wheezing. The first and second heart sounds were normal with no murmurs. The neurologic, genitourinary and gastrointestinal system examinations were unremarkable.

She had a hemoglobin level of 8 g/dl, and the 20 min whole blood clotting time (20WBCT) did not clot. Urinalysis (dipstick) showed hematuria 4+ while urine microscopy revealed numerous red blood cells. The white blood cell count and the platelet count could not be done. She was administered 10 ml of crotalidae polyvalent anti-venom intravenously over 10 min and vital signs were monitored for immediate allergic reactions. She also received anti-tetanic serum 1500 IU subcutaneously, intravenous dexamethasone 4 mg every 8 h, ceftriaxone 450 mg every 12 h, and local wound cleaning with an antiseptic solution. She was placed on intravenous normal saline 100 ml/h.

**Fig. 2:**
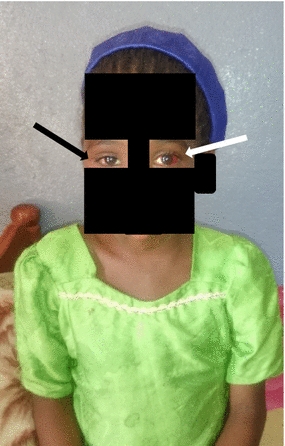
Resolution of right peri-orbital oedema (*black arrow*) and a lateral subconjunctival hemorrhage of the left eye (*white arrow*)

Twenty-four hours post admission, she had no signs of respiratory distress and facial edema was regressing. Anti-venom could not be continued due to medication stock out, and the nearest hospital with anti-venom was over 24 h away on a rough terrain. The next of kin was clearly informed of the management challenge and the inability to guarantee a safe referral, and he consented to continuing care in our health facility. Hematuria regressed from 4+ to 2+ and a control hemoglobin level was 5.0 g/dl. She was transfused 500 units of compatible and cross-matched whole blood on day 2 of hospitalization. The 20WBCT 6 h after the transfusion clotted. Her hematuria resolved completely 72 h post admission and her hemoglobin was 7 g/dl on day 7 of admission. She developed a fever on day 8 of hospitalization and was tested positive for Malaria infection, for which she received intravenous Artesunate. Following resolution of the edema, we noticed a left subconjunctival hemorrhage, with normal visual acuity and ocular movements in both eyes. She was discharged following total resolution of the swelling (Fig. 2) on day 10 post admission with hematinics but was lost to follow-up.

## Discussion

Snake bite is a neglected tropical disease [2] with over 314,000 bites and 7300 deaths each year in sub-Saharan Africa [12]. Estimates by the International Society of Toxicology revealed up to 6206 bites and 266 deaths each year in Cameroon, with most bites occurring in rural areas [1, 12, 13]. Children are at a higher risk for snake bites [2, 14]. The age adjusted morbidity from snakebites in Cameroon was 50 envenomations per 100,000 children aged less than 5 years and 400 envenomations per 100,000 children aged 5–14 years old [15]. This could be due to their curious nature which causes them to interact more with animals and because they are more likely to be found playing in the grass, fields, sand, water or places where snakes are easily found [3]. Also, due to their small size there is a greater amount of toxin injected per unit body mass making them more at risk to develop severe adverse effects, compared to adults [2, 3].

Furthermore, most snake bites are inflicted on the lower limbs, less often on the upper limbs and rarely on the face [13, 16]. Bites on the face have an increased risk of early systemic envenoming due to proximity to the heart when compared to limb bites [10]. Factors that favor severe envenomation following snakebite include; young age, ill health, bites on the trunk, neck and face, bites by large venomous snakes, bacteria in the mouth of the snake and mobilization and exertion following a bite [10, 17, 18]. Our patient met two of the above criteria, hence she was considered as a case of severe envenomation. A point of concern was the fact that the species of the snake could not be determined, but in about a third of reported snake bites, the assailing snake species was neither seen nor identified [13, 19]. Hence our diagnosis was made after seeing the fang mark and the patient’s report.

In Cameroon, there are over 150 species of snakes with only 32 being venomous. Of the venomous snakes, the most common is the *Crotalidae* species [20]. The *Dispholidus typus* (boomslang) is a *crotalidae* which is commonly found in trees and bushes in the North of Cameroon and its toxin induces coagulopathy and hemorrhage [20]. It’s likely that the offending snake might have been a boomslang as we identified a single puncture fang mark which is somewhat pathognomonic. The venom of boomslang has remarkable pro-coagulant effects, and activates factor II, IX and X of the coagulation cascade. This leads to a severe consumptive coagulopathy, which develops within 4–24 h following the bite. Bleeding usually presents as gingival bleeding, epistaxis, hematuria, hematemesis, melena, and subarachnoid or intracerebral hemorrhage in severe cases [21].

The main treatment for envenomation is the prompt administration of an appropriate dose of potent anti-venom [1, 22]. Snakes inject the same quantity of venom in adults as they do in children. Hence the same dose of anti-venom should be administered in adults and in children. The recommended dose of specific boomslang antivenom is 20 ml, diluted in an isotonic fluid and administered over 30 min. A second dose of 10 ml could sometimes be necessary [21]. Our patient received 10 ml of polyvalent *Crotalidae* anti-venom which had some antagonistic effects on the snake venom.

Also, the hematological set-up of the laboratory did not permit us to get a full hematological profile and she was followed up using the hemoglobin concentration and the 20WBCT. The 20WBCT is useful in confirming a coagulopathy and is used to monitor efficacy and adequacy of treatment [23]. The blood transfusion replaced the lost coagulation factors and helped to treat her coagulopathy even in the absence of adequate amounts of anti-venom. The lack of an accessible road network and the absence of a medicalized ambulance hindered safe referral of our patient and did not favor timely procurement of anti-venom.

Regardless of the fact that anti-venoms are the mainstay in the management of snake bite envenomation [1], the last 20 years have witnessed a crisis in the supply of anti-venoms in Africa. In Cameroon particularly, anti-venom serum has been out of stock at the central medicines supplier [Centre Nationale d’Approvisionement en Medicaments et Consomables Essentiel] (CENAME) for the past 3 years, hence the anti-venoms available are supplied by private pharmacies [24] often located in urban settings. This poses the extra problem of access to this emergency medication since majority of the cases occur in rural settings which offer better habitats to these reptiles as opposed to urban settings [2, 5]. Hence average travel distance to these procurement stores are generally not lower than a 100 kms [25].

These anti-venoms are often more expensive, costing up to 70,000 FCFA francs (about 140 United States Dollars (US$)) for a single vial, with some patients needing up to 14 vials for effective treatment [24, 26]. This amount is significantly higher than the guaranteed minimum wage in Cameroon which stands at 35,000 FCFA francs [27]. Most hospitals lack snake anti-venom or have a very limited supply, usually only a single vial, which is insufficient to fully manage a case of envenomation [26]. Meaning a patient who needs an adequate dose of snake anti-venom has to travel to an urban area and spend large sums of money to be able to get the snake anti-venom. In our case, the nearest town with a pharmacy which was guaranteed to have snake anti-venom was over 250 km away, along bad roads [25]. The causes of this shortage of anti-venoms are numerous, amongst which are: inefficient distribution channels within national health systems; ignorance of true anti-venom requirements due to poor epidemiological data on local snake populations; high cost of some products such that local health systems cannot afford; and lack of financial incentives to companies which used to supply anti-venoms to Africa [1, 28]. This decrease in the availability of anti-venoms has led to an increase in mortality and morbidity due to snakebites. Some neighboring countries have already taken steps to address the problem of snake anti-venom availability and distribution in their countries: Burkina Faso subsidizes anti-venom prices (up to 90%), causing the retail price to be 2500 FCFA (about US$ 5); Senegal forced every pharmacy in the country to stock permanently at least one vial of anti-venom; Togo has been supporting for 5 years the price of anti-venom by 60% in the public drug distribution system; Finally, Côte d’Ivoire has introduced the treatment of envenomation in the National Program of Universal Health Coverage that would be active by the end of 2015 [11]. This should act as an extra motivation to spur the Cameroonian government to be more proactive in addressing the problem of anti-venom availability by instituting health policies which encourage local production of anti-venoms and ensure distribution to all levels of the health system. One of the recommendations during the 6th international conference on snake bites and scorpion stings envenomation in Africa was to ensure the availability of anti-venoms through appropriate funding, defined after anthropological investigations on the acceptability of the price by the affected population. An equalization of anti-venom costs which will involve the state budget, support from local governments, companies employing workers at risk (such as agribusiness corporations), and health insurance groups [11].

General supportive therapy is an important component of snake bite management [29]. Depending on the specific needs of the bite victims, it usually combines: bed rest, wound dressing, reassurance, sedation, analgesics, prophylactic antibiotics, tetanus toxoid, steroids, intravenous fluids, wound debridement, limb elevation and observation [29–31]. Supportive therapy is simple, safe and effective in the treatment of snake bites, mostly those without severe systemic poisoning [29]. The shortage and high cost of anti-venoms in our setting [1] lays emphasis on the importance of supportive therapy in the management of snake bite victims. Our patient received a sub-optimal dose of anti-venom which complemented by effective supportive therapy resulted in a favorable outcome.

Other aspects of snake bite management include; patient education, management standardization, health personnel training and overall quality assurance. Their importance must not be under-estimated even in the presence of adequate amounts of specific anti-venoms [1]. In a study in Ghana, the institution of an intervention package for snake bite management, comprising: treatment protocol, staff training, monitoring of compliance and patient education, saw a 50% increase in snake bite admissions and a 90% decrease in mortality from snake bites, over a 15 month period [32]. A similar intervention could be expected to have a similar effect in our setting.

In Cameroon, the mortality from snake bites was 7.1% for children less than 5 years of age, and 3.2% for children aged 5–14 years old [15]. Despite this data suggestive of a high mortality in this patient’s age group, individual susceptibility to snake bite venom is however an important prognostic factor for snake bite victims. Our patient had several adverse prognostic factors: small size with a greater amount of toxin injected per unit body mass, bite to the face with an increased risk of early systemic envenoming due to proximity to the heart, administration of a sub-optimal dose of anti-venom and late arrival, 12 h after the bite. Going solely by the adverse prognostic factors coupled with the less than standard care offered, our patient had slim chances of survival. Nonetheless, she made a full recovery with near total remission of symptoms. This is a rare success story because envenomation following face snakebites in children carries a very high mortality rate as most of the children die even with optimal care [19]. This underlines the relevance of individual susceptibility to snake bite venom as an important prognostic factor for snake bite victims even in situations of adequate anti-venom administration and supportive therapy.

Patients in these areas should not have to rely on ‘luck’ to survive snake bite envenomation. This case report seeks to highlight the major shortcomings of local peripheral health facilities in terms of shortage of appropriate and effective anti-venoms, and poor national policies on snake bite management.

## Conclusion

Snake bites are a major public health problem in Cameroon with children being most at risk and having a poorer prognosis. Improved national public health policies are needed to assure availability of appropriate and effective anti-venoms at all levels of the health system. The institution of an intervention package involving: standard treatment protocol, staff training, compliance monitoring and patient education, will help decrease morbidity and mortality from snakebites.

## Abbreviations

20WBCT: 20 min whole blood clotting time
CENAME: Centre Nationale d’Approvisionement en Medicaments et Consomables Essentiel
CasE: Cameroonian Society of Epidemiology
IST: International Society of Toxicology
US$: United States Dollars
